# Stimulating the Stimulated Cortex—Frontocortical Anodal Electric Stimulation Combined With Closed‐Loop Acoustic Stimulation During Sleep Impairs Memory in Subjects With High Cognitive Ability

**DOI:** 10.1111/ejn.70266

**Published:** 2025-10-03

**Authors:** T. Hausdorf, A. Ferdinand, P. Koo‐Poeggel, M. Mölle, M. Bazhenov, L. Marshall

**Affiliations:** ^1^ Institute for Experimental and Clinical Pharmacology and Toxicology University of Lübeck Lübeck Germany; ^2^ Euroimmun Lübeck Germany; ^3^ Center of Brain, Behavior and Metabolism (CBBM) University of Lübeck Lübeck Germany; ^4^ Department of Medicine University of California San Diego La Jolla California USA; ^5^ University Hospital Schleswig‐Holstein, Campus Lübeck Lübeck Germany

**Keywords:** CLAS, memory consolidation, REM sleep, slow oscillation, tDCS

## Abstract

This study investigates the impact of simultaneous anodal transcranial direct‐current stimulation (tDCS) and closed‐loop acoustic stimulation (CLAS) during slow‐wave sleep on memory consolidation and neural oscillations. In this experiment, anodal tDCS was used to modulate cortical excitability, aiming to alter the brain state and investigate the resultant impact of CLAS‐induced effects on sleep electroencephalography and overnight memory consolidation. Twenty participants (aged 18–30) completed two experimental nights involving either CLAS alone or tDCS combined with CLAS (AmodCLAS). Offline detected spontaneous SOs were shifted toward negative potential values by AmodCLAS, SO duration was increased, and density decreased. AmodCLAS also decreased sleep efficiency and REM sleep in the second part of the night. Compared with CLAS alone, AmodCLAS failed to influence memory consolidation across all subjects. However, memory retention of participants with higher fluid intelligence (as measured by Raven's Advanced Progressive Matrices) was significantly decreased after AmodCLAS, together with a more pronounced negative shift of SO up‐state intervals, yet a weaker reduction in REM sleep. Our findings suggest that individuals with higher cognitive ability exhibit greater susceptibility to sleep‐based neuromodulation while possibly possessing greater resilience against sleep perturbance. This highlights the critical role of interindividual cognitive differences in shaping responsiveness to neuromodulation techniques and underscores the need for personalized approaches during such interventions.

## Graphical abstract

Can responsiveness to closed‐loop acoustic stimulation (CLAS) be modulated by anodal transcranial direct current stimulation (AmodCLAS) during sleep? Such induced differences in excitability may partially explain variability in CLAS efficacy. AmodCLAS affected offline detected slow oscillations, reduced sleep efficiency, and REM sleep. Retention was decreased in subjects with high fluid intelligence. The necessity to consider interindividual differences in neuromodulation studies and potential therapy is highlighted.

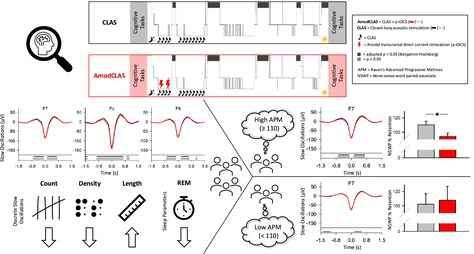

**Abbreviations**
AmodCLAS: anodal tDCS modulated closed‐loop acoustic stimulation
AN(C)OVA: analyses of (co)variance
APM: Raven's advanced progressive matrices
CLAS: closed‐loop acoustic stimulation
EEG: electroencephalography
EMG: electromyogram
EOG: electrooculogram
FPA: figural paired‐associate
NREM: non‐rapid eye movement
NSWP: nonsense‐word paired‐associate
PANAS: positive and negative affect scale
PFC: prefrontal cortex
RMS: root mean square
SO: slow oscillation
SSS: Stanford sleepiness scale
tDCS: transcranial direct‐current stimulation
WPA: word‐paired‐associate

## 1 Introduction

Sleep, especially slow wave sleep, has been associated with synaptic (Tononi and Cirelli 2014) and glucose homeostasis (Vallat et al. 2023), immune function (Besedovsky et al. 2019), circadian rhythmicity (Franken and Dijk 2024), and cardiovascular (Tobaldini et al. 2017) and respiratory activity (Schreiner et al. 2021), as well as memory consolidation (Rasch and Born 2013; Klinzing et al. 2019). Most recently, the specific role of slow wave activity toward growing problems of mental health was discussed (Chellappa and Aeschbach 2022). The relationships between these physiological systems are mostly bidirectional or even multidirectional, affecting activity from the level of molecules and cells to local networks and whole (brain) systems. Moreover, although for active systems, consolidation reactivation initiated in the hippocampus is most commonly emphasized, there is multiple evidence that the cortex is simultaneously active or may even take the lead in bidirectional interactions consolidation (Wagner et al. 2010; Rothschild et al. 2017; Klinzing et al. 2019; Chao et al. 2020; Ngo, Fell, et al. 2020; Feliciano‐Ramos et al. 2023).

Noninvasive brain stimulation during sleep is a means of probing the functional relevance and causality of brain rhythms (Campos‐Beltrán and Marshall 2017; Grimaldi et al. 2020). Moreover, it bears the potential of modulating brain rhythms and consequently associated functions.

In sleep, acoustic stimulation presents the predominant noninvasive brain stimulation approach. The application of acoustic stimulation synchronized to the sleep slow oscillation (CLAS and PPL methods) has led most consistently to enhancements in slow wave activity in humans. However, behavioral effects, i.e., on memory consolidation or the homeostatic SO function in facilitating postsleep learning, were less consistent (Wiethoff et al. 2014; Dyke et al. 2016; Horvath et al. 2016; Papalambros, Santostasi, et al. 2017; Ong, Patanaik, et al. 2018; Henin et al. 2019; Papalambros, Weintraub, et al. 2019; Malkani 2020; Koo‐Poeggel, Neuwerk, et al. 2022). Transcranial direct‐current stimulation (tDCS) affects neuronal excitability and neuronal plasticity (Jacobson et al. 2012; Jackson et al. 2016; Yavari et al. 2018; Ren et al. 2022), possibly by also influencing neuromodulatory tone (Adelhöfer et al. 2019; Mishima et al. 2019).

Notably, the mechanisms through which these stimulation modes exert an effect on brain activity differ. Weak electric stimulation modulates primarily cortical excitability and synaptic plasticity but also cortical–subcortical functional connectivity (Filmer et al. 2014; Bradley et al. 2022; Park et al. 2023). The mechanisms of closed‐loop acoustic stimulation (CLAS) synchronized to the sleep slow oscillation may primarily act through direct facilitation of the coupling of thalamo‐cortico‐hippocampal activity (Aksamaz et al. 2024).

For basic research but also for future clinical implementation, it is essential to disclose the reasons for such behavioral variability. Along with stimulation procedures, stimulation parameters, and composition of the subject group, brain state and interindividual differences pose to be decisive for the efficacy of stimulation on behavior, specifically on memory consolidation (Dedoncker et al. 2016; Dehnavi et al. 2021; Bradley et al. 2022; Alipour et al. 2024).

In the present study, we aim to investigate the differential response to CLAS on electrophysiological and behavioral measures at different levels of cortical activity, as induced by tDCS. Thus, anodal tDCS is used to simulate a brain state of increased frontocortical excitability and activity. On the assumption that hippocampal–frontocortical interactions are relevant for declarative memory consolidation during sleep (Klinzing et al. 2019), and anodal transcranial electric stimulation over the frontal cortex in many—although not all—studies previously facilitated memory consolidation during sleep (Marshall et al. 2004; for meta‐analyses, see Barham et al. 2016; Ladenbauer et al. 2017; Ketz et al. 2018), we hypothesized that the increase in cortical excitability by anodal tDCS over the dorsolateral prefrontal cortex (PFC) would support hippocampal–frontocortical communication, reflected in increased spindle activity and SO‐spindle coupling and, most importantly, in improved retention performance. Memory consolidation was not increased. However, individual differences in cognitive efficiency became apparent, and an unexpected effect on REM sleep was found.

## 2 Material and Methods

### 2.1 Subjects

Data of 20 subjects (14 females) between 18 and 30 years, right‐handed (as confirmed by the questionnaire of Annett handedness (Annett 1970), nonsmoking subjects with good knowledge of the German language participated in the study. Exclusion criteria were mental and neurological pre‐existing conditions, use of medication affecting the activation level or hormone balance, except for contraceptives, family history of epilepsy, metallic implants, shift work, and prior participation in studies employing similar cognitive tasks. On experimental days, subjects were instructed not to drink caffeine or alcohol after noon, and stressful events were to be avoided.

Studies were conducted at a sleep laboratory of the Center of Brain, Behavior and Metabolism, University of Lübeck. Initially, 47 subjects were recruited between October 2020 and May 2021 by using an online platform of the University of Lübeck and regional advertisements. Thirteen participants were excluded after the adaptation night due to poor sleep, and for the same reason, 10 subjects were excluded after the first experimental night; in particular, the amount of time in N3 was below 20 min. Twenty‐four participants finished all three nights, whereby three subjects were subsequently excluded due to consistent awakening in the stimulation night, and/or the corresponding number of slow oscillations (sham‐ or stim triggers) was below 140, and one subject was excluded due to technical issues. Informed written consent was given by all participants before participation. Participants were reimbursed for their participation. Approval from the local ethics committee of the University of Lübeck in accordance with the Declaration of Helsinki was given for the study.

### 2.2 Procedure

Subjects underwent an adaptation night in the laboratory, subsequently two experimental sessions for sleep, and a subsequent daytime session to test for cognitive abilities. The two experimental conditions were pseudorandomized and counterbalanced across subjects. Shortly before lights‐out (~22:30 h), delivery of acoustic stimulation and a test for successful tDCS delivery were performed. As in the experimental sessions, subjects were awoken after 8 h from light non‐rapid eye movement (NREM) sleep. Exemplary tasks for learning and recall were given before and after sleep. The adaptation night also served to control adequate SO detection and to assess the optimal volume for acoustic stimulation in N3 sleep. The delta–theta ratio and delay time between SO negative half‐wave detection and acoustic stimulation for the experimental setup were calculated offline. The adaptation session for female participants without hormonal contraceptives was scheduled at the beginning of their luteal phase (between the 15th and 21st day of the cycle). Adaptation and the first experimental session were separated by a minimum of 2 and a maximum of 7 days.

**FIGURE 1:**
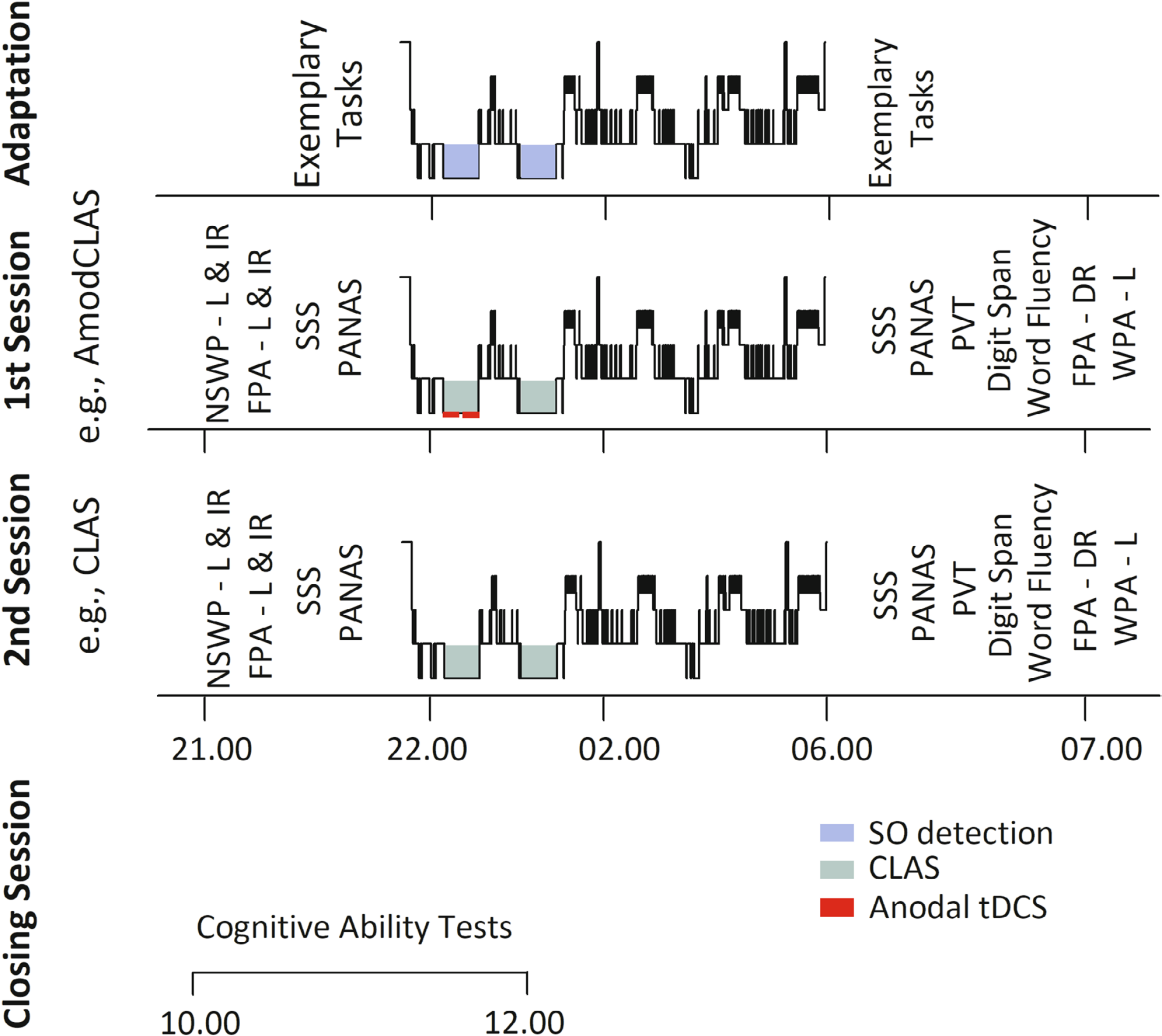
Time course of the experimental procedure. AmodCLAS: anodal transcranial direct current stimulation (tDCS) modulated closed‐loop acoustic stimulation (CLAS); DR: delayed recall; FPA: figural paired‐associate task; IR: immediate recall; L: learning; NSWP: nonsense‐word paired‐associate; PANAS: positive and negative affect scale; PVT: psychomotor vigilance test; SO, slow oscillation; SSS: Stanford sleepiness scale; WPA: word pair‐associate task.

The two experimental nights were separated by at least 14 days (28 days for cycling female subjects). In the experimental sessions, either anodal tDCS together with CLAS (AmodCLAS, anodal modulated CLAS) or CLAS alone (CLAS) was performed. For both, subjects arrived at 20.00 h. After electrode application, subjects learned two declarative tasks. Psychometric control parameters were assessed shortly before going to bed. Lights off and awakening were as in the adaptation night. Forty minutes after awakening, recall of the two declarative tasks and learning on a third, hippocampus‐dependent encoding task occurred. Psychometric control tasks were performed, as indicated in Figure 1.

### 2.3 Memory Tasks

Two declarative memory tasks (figural paired‐associate [FPA] and nonsense‐word paired‐associate [NSWP]), as well as one task to assess encoding performance (word‐paired‐associate, WPA), were conducted. The FPA was previously described (Koo et al. 2018; Koo‐Poeggel et al. 2022). In brief, in the evening, subjects were presented in two trials with two lists of 16 figure pairs (geometric and nongeometric lines and shapes) that differed in order to learn. Each pair was displayed on a screen for 4 s, with an interstimulus interval of 500 ms. In the subsequent immediate recall, a figure was shown, and subjects were to select via mouse click the matching figure from seven others. There was no time limit, and feedback was provided. The correct pair was presented on the screen for 2 s. In the morning, delayed recall, but without feedback, was conducted as in the evening, with figures presented in a different order. Across subjects, lists of items were pseudorandomized according to a Latin Square.

The NSWP task consisted likewise of 16 nonword pairs of two syllables each (e.g., GEMKIN and KRASUM), which were presented for 3 s in the identical fashion as the figural pairs. Immediate and delayed recall were conducted as in the FPA task, but with only one learning trial. Figures and nonsense words were adapted from Sturm and Willmes (1999).

Learning on the FPA and NSWP was defined as the number of correctly recalled items at immediate recall (mean of both trials for FPA). Retention performance was defined as the percentage of correctly recalled items at delayed recall relative to learning performance.

Furthermore, encoding performance in the morning after nocturnal sleep was measured by WPA, adapted from Koo‐Poeggel et al. (2022). The test consisted of a total of 80 semantically unrelated pairs of German nouns, a cue and a target word. Both abstract (e.g., friendship) and concrete nouns (e.g., fox) were used. Pairs were presented for 3 s each, with an interstimulus interval of 500 ms. At immediate recall, subjects responded to each cue word by speaking the target word into a microphone, without time pressure. This procedure of encoding and immediate recall was repeated three times. No feedback was given. Each trial consisted of a different pseudorandomized order. Audio files were analyzed by a blinded person. Data from one participant were omitted because of a technical issue. Learning performance on the WPA was defined as the percentage of the number of correct words recalled on each of the three trials relative to the total number of word pairs.

### 2.4 Psychometric Control Data

The positive and negative affect scale (PANAS) (Watson et al. 1988) and Stanford sleepiness scale (SSS) (Hoddes et al. 1973) were completed just before the lights out in the evening and before cognitive testing in the morning. In the morning, a 5‐min psychomotor vigilance task (PVT) (Roach et al. 2006) was additionally conducted to check the attention and alertness of the participants.

In the morning after cognitive testing, the Regensburger verbal fluency test (RWT) (Aschenbrenner and Lange 2001) was conducted to assess semantic and phonematic word fluency. The digit span forward (DSF) and backward (DSB) assess the span capacity and ability to manipulate working memory, respectively. Both digit span tests are part of the Wechsler Adult Intelligence Scale (Wechsler 1997). At the end of the morning session, subjects reported whether they had felt or heard anything during the night and whether they thought the session was with or without (electric) stimulation.

### 2.5 Cognitive Ability

A session to assess cognitive ability, specifically general fluid intelligence (APM, Ravens's Advanced Progressive Matrices; Raven et al. 2010) and memory quotient score (Bäumler 1974), took place from 10 to 12 a.m., 2–7 days following the last experimental night. In brief, in the APM, for Set I, subjects were given 10 min; this subtest served to familiarize participants with the test type and was not scored. For Set II, subjects had a 30‐min time limit. In the test, given patterns are to be completed; the participant must select one of eight options in each case. The number of correct answers was converted into a weighted score. The German Bäumler's Lern‐ und Gedächtnistest (Bäumler 1974) contains six subtests with individual time limits. Subtests' raw scores were transformed into weighted *t*‐value points from which a general memory quotient is obtained.

### 2.6 EEG Acquisition

EEG from 13 sites according to the international 10–20 system (F7, F8, [F3 and F4 used for anodal electrodes], Fz, C3, Cz, C4, P3, Pz, P4, O1, O2, M1, and M2) was amplified and recorded between 0.03 and 80 Hz (50‐Hz notch filter) at a sampling rate of 500 Hz (LiveAmp, Brain Products GmbH, Gilching, Germany). Cz served as the reference and AFz as the ground. EEG data were re‐referenced to linked mastoids. The bipolar recordings VEOG, HEOG, and EMG were implemented for standard polysomnography and artefact detection. Electrocardiography (ECG) was recorded from the right arm and anterior axillary line at the level of the fifth intercostal space (V5) from the 12‐lead ECG configuration and amplified between 1 and 25 Hz with the same amplifier. Ag‐AgCl sintered ring electrodes (Multitrode, EASYCAP GmbH, Herrsching, Germany) were attached using adhesive EEG paste. Impedance was kept below 5 kΩ for EEG, HEOG, and VEOG and below 10 kΩ for EMG and ECG. For analyses, the EEG data were re‐referenced to linked mastoids using Brain Vision software (Brain Products GmbH, Gilching, Germany).

### 2.7 SO Detection and CLAS

The basic principle of SO detection and CLAS stimulation was as described previously (Ngo, Martinetz, et al. 2013; Koo‐Poeggel et al. 2022). In brief, the adaptation data were used to determine an individual threshold for the prefrontal delta/theta ratio and to calculate an individualized SO delay time, defined as the average interval between the EEG SO negative peak and the subsequent positive, for use in CLAS stimulation. We used a customized script running under Spike 2 software version 9 (Cambridge Electronic Design, UK) together with a sequencer in the Power1401 mk2 to monitor the Fpz signal in real time. Fpz was referenced to linked mastoids to detect SO online, with the EEG signal filtered between 0.25 and 4 Hz (Digitimer, Hertfordshire, UK) at a sampling rate of 200 Hz. The delta–theta ratio was continuously calculated to assist in determining NREM sleep using the same electrode sites but filtered between 0.25 and 45 Hz (Digitimer, Hertfordshire, UK).

CLAS was triggered when two criteria were met (Ngo, Martinetz, et al. 2013): (1) The EEG signal exceeded an adaptive amplitude threshold in negativity (updated every 0.5 s, based on the EEG amplitude of the preceding 5 s), which was initially set at −80 μV. (2) The delta/theta ratio was above the individual threshold for NREM sleep. From the data of the adaptation, the delta/theta ratios of each 30‐s sleep epoch were calculated. Two histograms were generated containing the number of epochs per delta/theta ratio for either wakefulness and REM sleep or NREM sleep. The optimal delta/theta ratio for an individual subject was defined visually as the ratio at which the numbers of Wake/REM epochs were of relatively low value and the number of NREM sleep epochs had strongly increased. The average ratio was around 23.6 (±1.8) (cp. Aksamaz et al. 2024). The CLAS protocol was adapted from Ngo, Martinetz, et al. (2013). In brief, in the AmodCLAS condition, CLAS was manually activated by the investigator after at least 4 min of stable N3 sleep for the first deep sleep cycle, 2 min of N3 for any subsequent sleep cycle. If, as in most instances, tDCS was performed parallel to CLAS, electrical stimulation was manually initiated first. Acoustic stimuli were only released after complete attenuation of electric stimulation artefacts (approximately 30 s). CLAS was also terminated shortly before the end of tDCS (approximately 30 s) to avoid artefacts. In the sham‐tDCS condition, CLAS was initiated after ~4.5 min (4 min + ~30 s) of stable N3 for the first sleep cycle and ~2.5 min (2 min + 30 s) for any subsequent sleep cycle, introducing CLAS at closely comparable periods as for AmodCLAS.

Acoustic stimulation consisted of two bursts of pink 1/f noise of 50‐ms duration, including 5 ms of each rising and falling time. The first burst was delivered after the individualized delay time from the EEG SO negative half‐wave to shortly before the SO positive peak, measured from the mean of offline SOs detected in the adaptation night. The second burst came after a fixed interval of 1075 ms. A pause of at least 2.5 s followed before the next SO was detected. On delivery of the acoustic stimuli, triggers were sent to the EEG device (LiveAmp, Brain Products GmbH, Gilching, Germany). Sounds were played bilaterally through in‐ear phones (MDR‐EX15LP, Sony Germany) worn by the subject throughout the night. Volume was initially set to 35 dBA and was increased every five to 10 triggers, with a target of 49 dBA. When the subject showed signs of arousal or shifted toward lighter sleep, CLAS was terminated, and the target volume was readjusted. CLAS was limited to the first 4 h time in bed. We aimed for the same amount of CLAS in the second session as had been applied in the first experimental session.

### 2.8 Transcranial Direct Current Stimulation

Two parallel circuits were used to deliver anodal tDCS bilaterally to sites F3 and F4 (each 9 cm^2^, Neuroconn, Ilmenau, Germany) with return electrodes (each 14 cm^2^) positioned between P3 and O1, and P4 and O2, respectively, and attached using conductive paste (Ten20 Conductive, Weaver and Company, Aurora, USA). The two stimulator devices (NeuroConn, DC‐Stimulator Plus) were in a monitoring room adjacent to the subject. A ramp up and down of 8 s with a maximum current intensity of 800 μA per circuit was used. Impedance was kept below 2 kΩ.

In the condition with tDCS (AmodCLAS), the experimenter aimed to apply current twice for 15 min, with a 5 min break within the first half of the night (first 4 h of sleep). TDCS began after 4 min of stable N3 sleep of the first deep sleep cycle and 2 min of stable N3 sleep of any subsequent sleep cycle. Furthermore, a stable delta–theta ratio above 20 at Fpz (short pauses of a maximum of 5 s were accepted) was required. When subjects awoke or switched to a lighter sleep stage, tDCS was immediately terminated. TDCS or sham‐tDCS during N3 continued throughout the night until subjects had received 30 min in total. In CLAS, only 30‐s epochs of tDCS were applied. In the adaptation session, CLAS was given once only as a probe. The tDCS protocol was based on findings by Monte‐Silva and colleagues (Monte‐Silva et al. 2013) and the ~30‐min average duration of N3 during the first sleep cycle.

### 2.9 EEG Analyses

For polysomnography, sleep stages were classified with SleepPilot (https://github.com/xuser/SleepPilot, v0.9.4‐beta) by two independent researchers blinded to the condition, using EEG recordings from Fz, C4, Pz; VEOG, HEOG, and EMG, and 30‐s EEG epochs. Wake, N1, N2, N3, and REM sleep were determined according to the guidelines of the American Academy of Sleep Medicine (Berry et al. 2012). In addition, moving arousals and artifacts were marked. The time frames of tDCS were marked subsequently for further EEG analyses.

For offline identification of spontaneous SOs throughout NREM sleep N2 and N3 (time‐locked to the negative half‐wave peak) for both experimental conditions, the procedure of Mölle and colleagues (Mölle et al. 2011) was adopted. In brief, from the bandpass filtered 0.5‐ to 3.5‐Hz EEG signals, negative and positive peak potentials were detected from all intervals with zero‐crossings separated by 0.75–2 s. Subsequently, mean amplitude thresholds were determined for all negative and negative‐to‐positive peak potentials, from thresholds set at 1.25 times the mean negative or negative‐to‐positive peak potentials, respectively. A SO was defined when, for successive negative and negative‐to‐positive peak potentials, amplitudes surpassed the respective thresholds.

Discrete spindles were similarly detected from the low‐pass filtered and downsampled EEG data. Spindle detection was adopted from Mölle et al. (2011). In brief, individual fast (12–16 Hz) and slow (9–12 Hz) spindle peak frequencies were identified from power spectra at all channels throughout all artefact‐free NREM sleep periods (N2 and N3). The mean slow spindle peak frequency was 10.8 ± 0.2 Hz, and the mean fast spindle peak frequency was 13.5 ± 0.1 Hz. A FIR band‐pass filter with a width of 3 Hz centered on the detected individual peak frequencies was then applied to the EEG signals. An RMS representation of the filtered signal was calculated using a sliding window of 0.2 s and a step size of one sample. Subsequently, RMS data were smoothed with a 0.2‐s‐size sliding window. Time frames were considered as spindle intervals if the RMS signal exceeded a threshold of 1.5 SD of the bandpass filtered signal for 0.5–3 s, and at least one sample point was above a threshold of 2.5 SD of the bandpass filtered signal. Detected spindles were merged if the intervening interval was < 0.5 s, and the total RMS superseding the 1.5 SD threshold was < 3 s. Spindle count, density (i.e., number of spindles per 30 s), mean peak‐to‐peak amplitude, mean length, and mean spindle RMS throughout NREM sleep were analyzed. For both CLAS and AmodCLAS sessions, thresholds for offline detection of spontaneously occurring SOs and discrete spindles throughout NREM sleep were derived from the CLAS session.

To assess stimulus‐related SO, slow and fast spindle evoked activity, time‐locked to the first stimulus of CLAS, EEG signals were first low‐pass filtered at 35 Hz (FIR filter, −3 dB at 32.0 Hz) and downsampled to 100 Hz. Windows of −1 to 3 s time‐locked to the first stimulus were extracted from artefact‐free epochs in N2 and N3 and averaged. For spindle activity, before extracting the 4‐s windows time‐locked to the acoustic stimuli, signals were bandpass filtered with a width of 3 Hz around individually determined spindle peak frequencies (within 9–12 Hz for slow spindle activity; within 12–16 Hz for fast spindle activity), and for each sample point, the root mean square was calculated and smoothed (RMS; time window: 0.2 s).

Power spectra were determined by fast Fourier transformations using a Hanning window with 4096 data points (8.2 s) and 50% overlapping windows, resulting in a frequency resolution of 0.12 Hz, calculated frequency bands for SO (0.5–1.5 Hz), delta (1.5–4 Hz), slow wave activity (0.5–4 Hz), theta (4–9 Hz), slow spindle (9.5–12.5 Hz), and fast spindle (12.5–15.5 Hz) bands. Bands of EEG power were logarithmized prior to statistical analyses to obtain normality. Data analyses were performed using Spike2 (Cambridge Electronic Design, Cambridge, England).

### 2.10 Statistical Analyses

Statistical comparisons of means were based on data averaged per subject. Differences between conditions in offline detected spontaneous SOs time‐locked to the negative half‐wave peak, as well as stimulus‐related SO and spindle RMS responses time‐locked to the first acoustic stimulus, were tested with running paired nonparametric tests (Wilcoxon). The Benjamini–Hochberg procedure was used to control the false discovery rate. Note that both uncorrected results and results corrected for false discovery rate are shown for comparative purposes, since similar studies on CLAS have often depicted uncorrected *p*‐values (Ngo, Claussen, et al. 2013; Ngo, Martinetz, et al. 2013; Ong, Lo, et al. 2016). Stimulus‐related time‐locked SO responses were baseline‐normalized using the time window −0.99 to −0.01 ms as a reference, unless otherwise reported.

Statistical analyses of the properties of offline detected spontaneous oscillatory events (SOs and spindles) were conducted by repeated measures analyses of variance (ANOVA) with the factors condition, COND (levels: CLAS and AmodCLAS), and corresponding scalp topography, TOPO (levels: F7, Fz, F8, C3, Cz, C4, P3, Pz, P4, O1, and O2). In our analysis (Figure 4), we distinguished between spontaneously occurring SOs during (i) the entire NREM sleep period and a subgroup occurring (ii) specifically during tDCS periods in the “AmodCLAS” experimental condition (or during the pseudo‐tDCS‐on phase in the “CLAS” experimental condition (Figure S2).

Retention performance was analyzed using factors COND (CLAS and AmodCLAS) and TIME (immediate recall, delayed recall) in a repeated measures ANOVA for all memory tasks separately. To assess group effects, APM and MQ scores were entered as covariates into a repeated measures ANCOVA. For further behavior analysis dependent upon APM, a group factor was included reflecting a high‐ and low‐APM group (median = 110). Differences in retention performance (delayed recall—immediate recall) between conditions (CLAS and AmodCLAS) or modification in retention performance between APM groups were assessed by dependent and independent paired comparisons, respectively. An ANCOVA was similarly conducted for the post hoc assessment of differences in the SO positive half‐wave of offline detected SOs at the three frontal locations (F7, Fz, and F8) within the interval ±0.3 to 0.6 s before and after the negative half‐wave peak. For the morning WPA learning task, a one‐way repeated measures ANOVA with the factor Learning TRIALS (L1, L2, L3) was employed.

All ANOVA/ANCOVA results are Greenhouse–Geisser corrected to compensate for any violation of sphericity (uncorrected degrees of freedom are given). Distributions within the limits of normality were tested using Shapiro–Wilk, skewness and kurtosis values, and Q‐Q plots. Paired comparisons were conducted by *t*‐tests for normally distributed variables and Wilcoxon signed‐rank tests where assumptions of normality were violated (*p* < 0.05 on the Shapiro–Wilk test). Unless reported otherwise, all values are given as mean ± SEM. A value of *p* < 0.05 was considered significant. Exploratory behavioral and polysomnography analyses were not subject to multiple comparison correction.

Statistical analyses on stimulus‐related, time‐locked responses to CLAS were conducted with Jamovi (v2.3.21) and R/RStudio (Version 2024.04.1+748), and analyses of behavior, discrete events, and EEG power were conducted with SPSS (IBM SPSS Statistics, version 22.0, IBM, Armonk, NY, USA).

## 3 Results

The study involved 20 subjects aged 18–30, all nonsmokers, with good knowledge of the German language, who underwent CLAS or CLAS combined with, i.e., modulated by anodal tDCS (AmodCLAS). After an initial adaptation night to adjust sleep detection and stimulation conditions, participants completed two experimental sleep sessions followed by daytime cognitive tests. Tasks presented to subjects involved immediate and delayed recall of paired associates to test memory consolidation across sleep sessions and encoding performance in the morning. Data collected included EEG recordings, memory performance, psychometric data (e.g., affect and vigilance), and cognitive ability (see Section 2 for details).

### 3.1 AmodCLAS and Behavior

**FIGURE 2:**
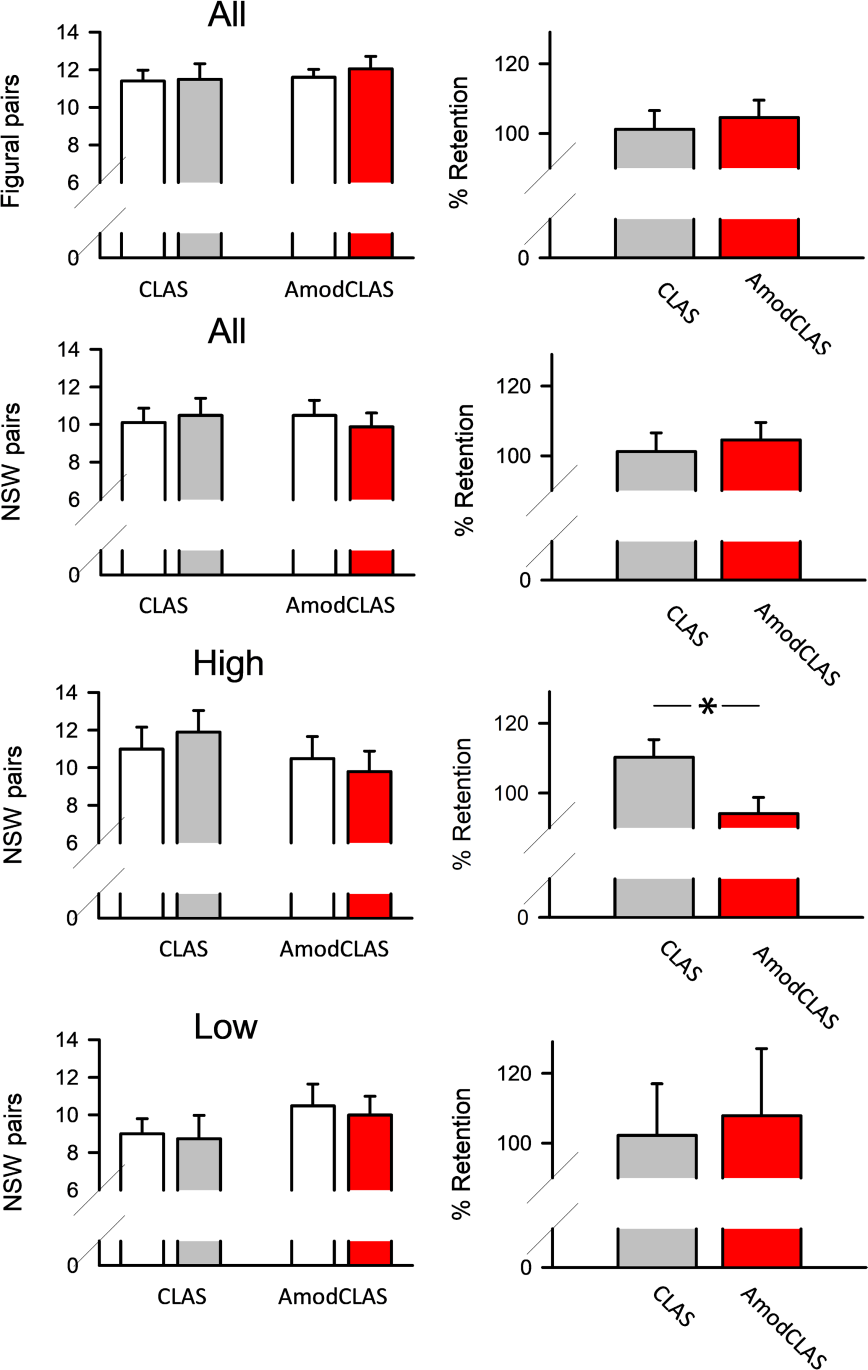
Retention performance for the figural‐paired task (*N* = 19) and nonsense word (NSW) paired task (*N* = 18). Left: Learning (open bars) and recall performance (filled). Right: Percent retention performance, with learning performance set to 100%. Results across all subjects are shown in the top two rows for CLAS and AmodCLAS conditions. The bottom two rows depict results for the subjects scoring high (*N* = 10) and low (*N* = 8) on the advanced progressive matrix (APM) scale. *p < 0.05, Wilcoxon signed‐rank test.

Across all subjects, no significant differences in any of the cognitive tasks were observed (*F*(1,19) < 0.344, *p* > 0.5). Although our intent was to simulate interindividual differences in the response to CLAS by tDCS alone, previously trait‐like features affected stimulation efficacy. Thus, we considered APM score and memory quotient scores as potential modulators of tDCS efficacy in the present study. Considering memory quotient scores as a covariate did not affect CLAS efficacy. However, the efficacy of AmodCLAS on the NSWP task performance interacted significantly with the APM score, *F*(1,16) = 19.809, *p* < 0.001, for COND × APM. NSWP performance of subjects with high, but not with low, APM was affected by AmodCLAS, revealing a performance decrement (Figures 2 and S1).

### 3.2 Modulation of EEG Activity by Anodal tDCS

**FIGURE 3:**
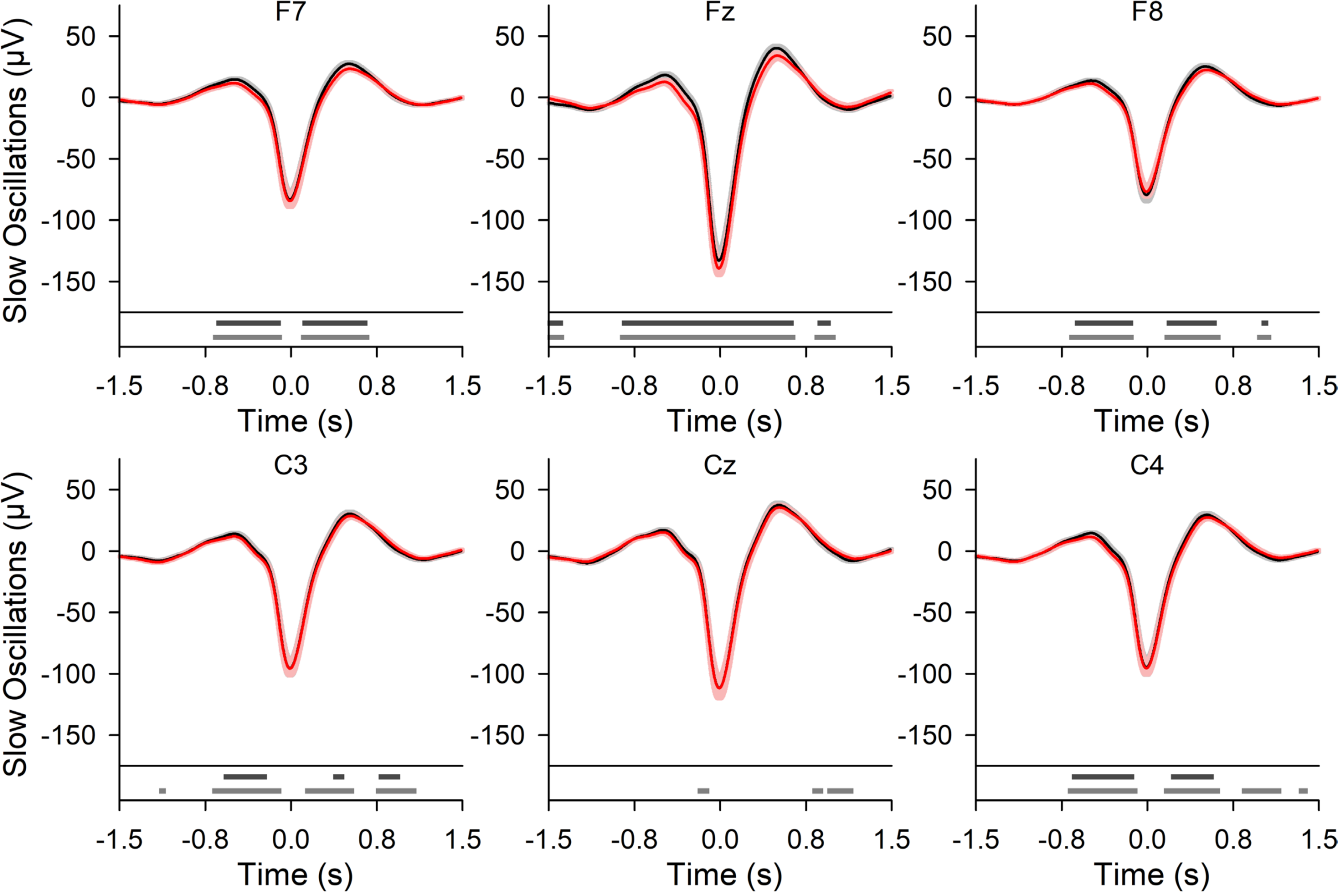
For all offline detected slow oscillations (SO) throughout NREM sleep, the effect on closed‐loop acoustic stimulation (CLAS) of anodal tDCS (AmodCLAS). Grand mean waveforms (± SEM) of spontaneous SO at locations F7, Fz, F8, C3, Cz, and C4 for CLAS (black) and AmodCLAS (red). Bottom diagrams: Horizontal bars indicate time points of significant differences (Wilcoxon, *p* < 0.05) without (gray) and with correction for false discovery rate (*p* < 0.05, dark gray). *N* = 20. Averages per subject consisted of 609.8 ± 12.2 (607.0 ± 14.9) epochs for CLAS (AmodCLAS).

In contrast to the failure of AmodCLAS to produce an effect across all subjects on memory consolidation and learning, sleep EEG and polysomnography were affected by AmodCLAS. Figure 3 reveals that epochs of offline detected SOs were shifted slightly, yet significantly, toward negative values. At Fz, both the SO negative peak and the time windows of preceding and following positive up‐states were affected. At other topographic locations, only SO slopes adjacent to the negative peak potential and potentials reflecting the up‐state positivity were significantly shifted toward more negative values.

**FIGURE 4:**
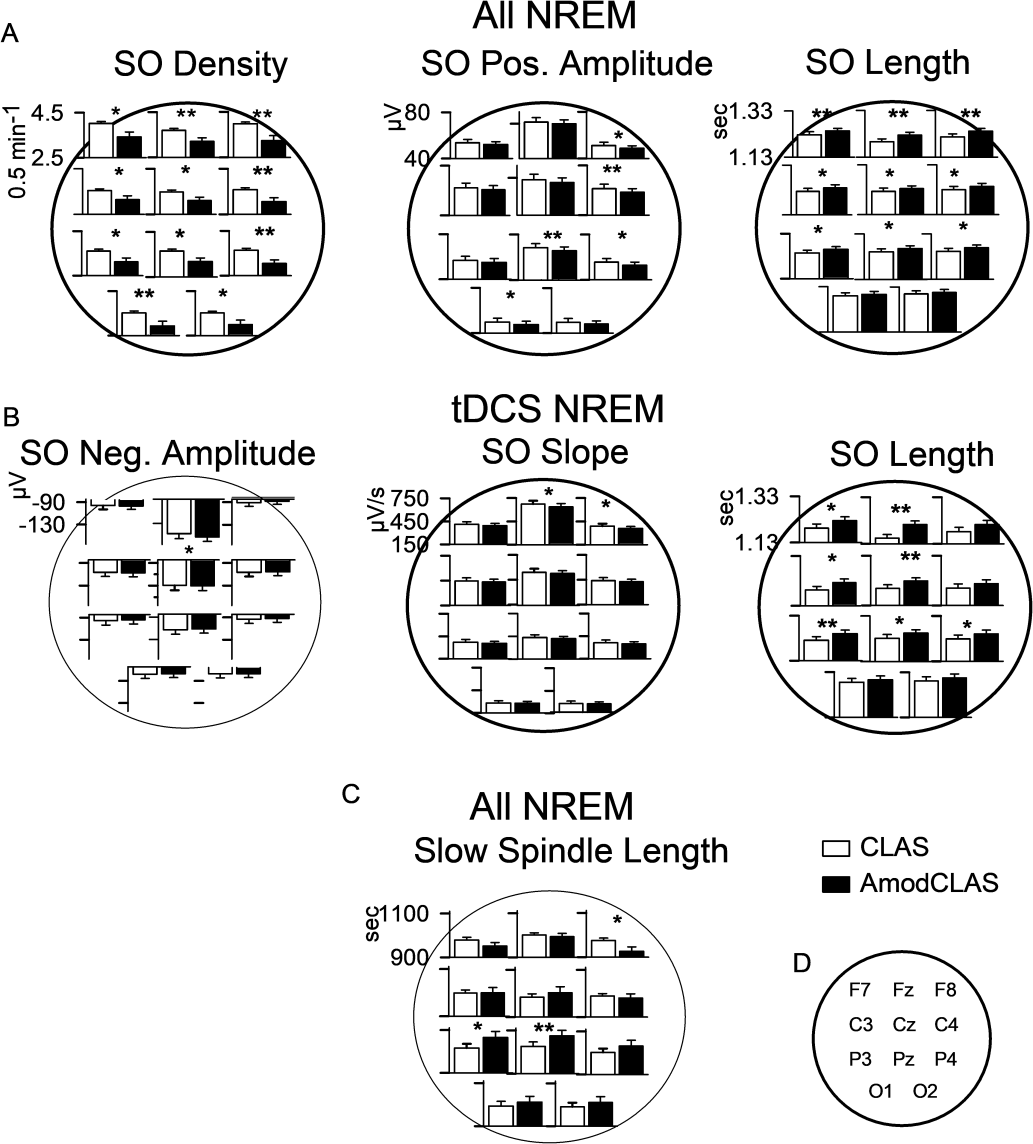
Effects on discrete SO and spindle measures of closed‐loop acoustic stimulation (CLAS) and AmodCLAS. Measures revealing significant condition differences for discrete SOs (A, B) in CLAS (white bars) and AmodCLAS (black bars) throughout NREM sleep (A), and during the acute tDCS time period (B) are shown, as well as significant effects for discrete spindle measures (C). Large, thick circle lines indicate a significant Condition main effect. Large thin circle lines indicate a significant Condition × Topography interaction. *p < 0.05, **p < 0.01 for post hoc tests. *N* = 20. *Note:* In Panel A, the SO positive amplitude range for Fz and Cz is 50–90 μV, rather than 40–80 μV. For all other diagrams, major tick labels are the same for all topographies, although the data range may be shifted.

Figure 4 reveals significant changes in properties of offline detected spontaneous SOs and spindles during the entire NREM sleep period, as well as specifically during the tDCS or pseudo‐tDCS‐on phase, respectively. Throughout NREM sleep, count, density, positive half‐wave amplitude, and length of discrete SO events were modified by AmodCLAS compared with CLAS (main effect of SO count: *F*(1,19) = 12.319, *p* = 0.002; density: *F*(1,19) = 14.367, *p* = 0.001; positive peak amplitude: *F*(1,19) = 7.038, *p* = 0.016; and length: *F*(1,19) = 7.744, *p* = 0.012). Although AmodCLAS decreased SO count, density, and positive half‐wave amplitude, it significantly increased SO length. During the anodal tDCS‐on periods, the length of SO events was similarly increased (*F*(1,19) = 9.960, *p* = 0.005 for the main effect; with a tendency toward topographical differences, *F*(10, 190) = 2.363, *p* = 0.063 for COND × TOPO interaction). In addition, the down‐to‐up‐state SO slope was shallower with AmodCLAS (*F*(1,19) = 5.401, *p* = 0.031, main effect of COND), tending to be stronger at frontal locations (*F*(10, 190) = 2.104, *p* = 0.082, interaction COND × TOPO). The negative SO peak amplitude was increased (more negative) at Fz (*F*(10, 190) = 3.472, *p* = 0.009, interaction COND × TOPO; Figure 4B). Spontaneous SO count, density, and SO positive peak amplitude were not changed significantly during the anodal tDCS‐on periods (*p* > 0.05). Throughout NREM sleep, the length of discrete slow spindle events changed in opposing directions at anterior versus posterior locations (*F*(10, 190) = 5.221, *p* = 0.001, COND × TOPO). At P3 and Pz, slow spindle length significantly increased but decreased at F8 by AmodCLAS (Figure 4C). Properties of discrete fast spindle events did not significantly differ between CLAS and AmodCLAS, nor did any other discrete slow spindle parameter.

Next, we determined whether the increased SO length could be attributed to positive versus negative SO halfwaves (SOHW). Since SOs are pronounced fronto and fronto‐central, analyses on SOHWs did not include the two occipital regions. Across all subjects, both positive and negative SOHWs were longer for AmodCLAS (Positive: *F*(1,19) = 5.679, *p* = 0.028; Negative: *F*(1,19) = 12.750, *p* = 0.002; *F*(8,152) = 3.417, *p* = 0.011 for COND × SOHW × TOPO; Figure S3). To obtain a closer differential topographical picture, we tested for differences in the ratio of positive to negative SOHW at all locations. The ratio was significantly larger for AmodCLAS, due to significantly longer negative SOHW relative to positive SOHW at Fz (*t*(19) = −14.382, *p* < 0.001; *F*(8,152) = 121.583, *p* < 0.001 for COND × TOPO, *F*(1,19) = 32.967, *p* < 0.001 for COND).

**FIGURE 5:**
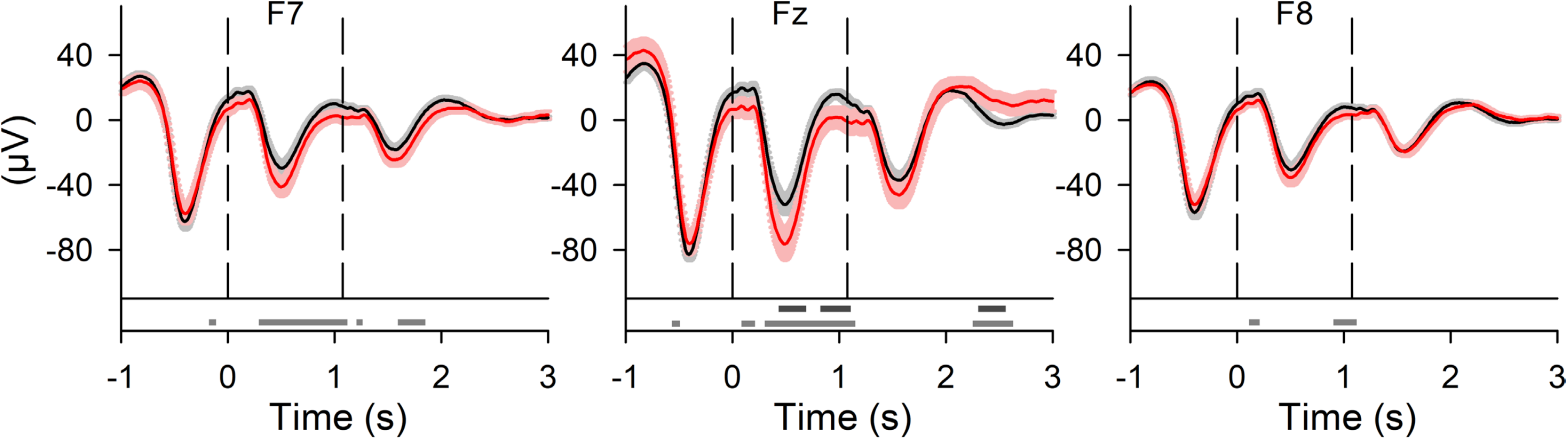
Stimulus‐locked EEG responses to acoustic stimuli in CLAS and AmodCLAS. Top diagrams: Grand mean waveforms (± SEM) of stimulus‐locked responses at locations F7, Fz, and F8 for CLAS (black) and AmodCLAS (red). Data are not baseline normalized. Bottom diagrams: Horizontal bars indicate time points of significant differences. Wilcoxon signed‐rank test, without (gray) and with (dark gray) correction for false discovery rate, *p* < 0.05. *N* = 19.

**FIGURE 6:**
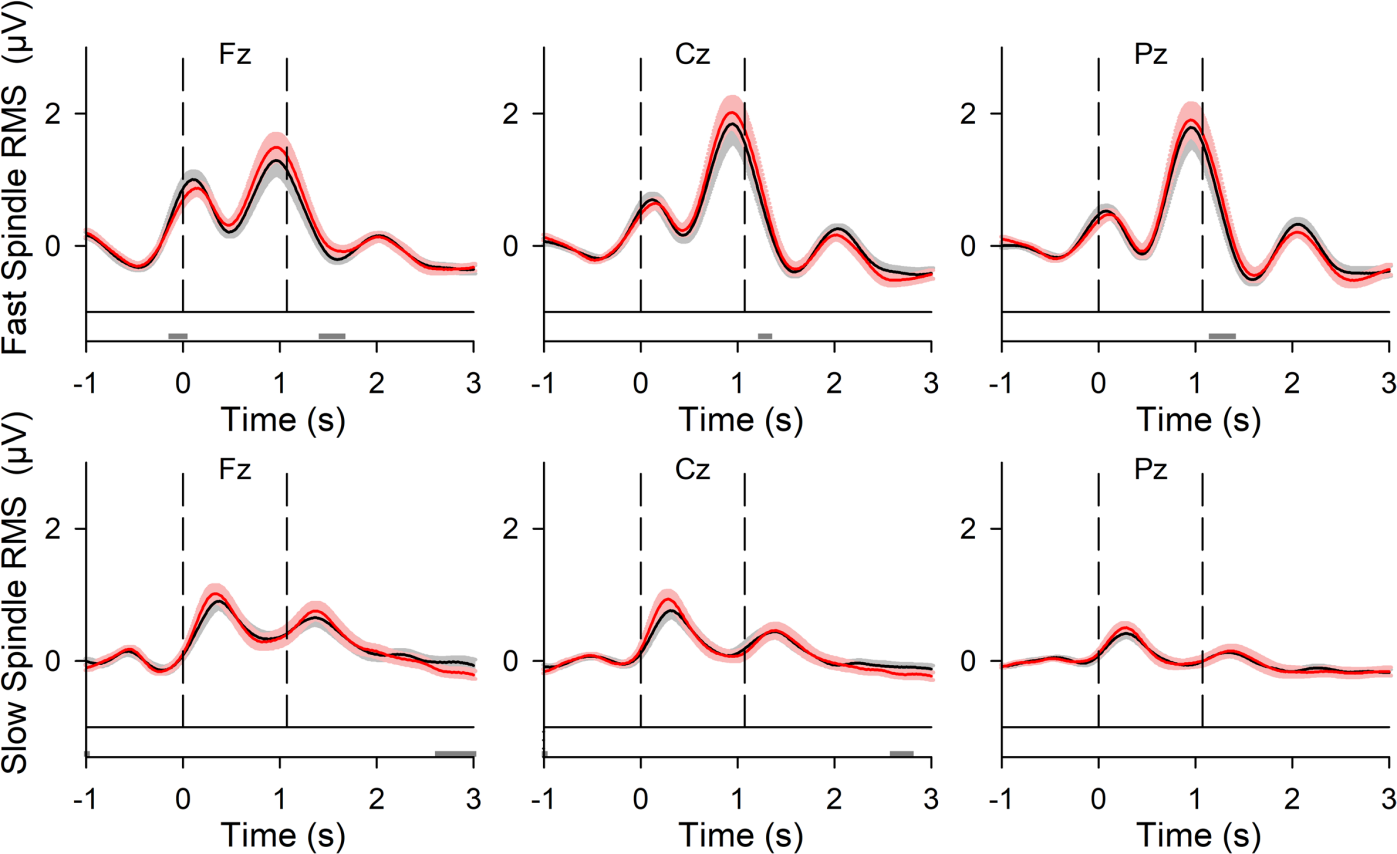
Stimulus‐locked spindle root mean square (RMS) to acoustic stimuli in CLAS and AmodCLAS. Top diagrams: Grand mean waveforms (± SEM) of stimulus‐locked responses at locations Fz, Cz, and Pz for CLAS (black) and AmodCLAS (red). Baseline normalization occurred over −0.99 to −0.01 s. Bottom diagrams: Horizontal bars indicate time points of significant differences. Wilcoxon signed‐rank test, *p* < 0.05, uncorrected. *N* = 19.

As expected, acoustic stimuli of both conditions generated a stimulus‐related evoked response (Figure 5). For comparison, Figure S4 shows data from another study (Koo‐Poeggel et al. 2022) comparing CLAS to a sham condition. In the present study, AmodCLAS shifted the stimulus‐related EEG response significantly toward more negative potential values after the first stimulus and around 2.5 s poststimulus significantly toward more positive EEG values at Fz (Figure 5). Figure S5 shows the stimulus‐related EEG response over a broader topographical distribution for baseline‐normalized data. Stimulus‐related fast and slow spindle RMS are presented in Figure 6. None of the qualitative differences in baseline‐normalized time‐locked spindle RMS remained significant after correcting for multiple comparisons (Figure 6).

**FIGURE 7:**
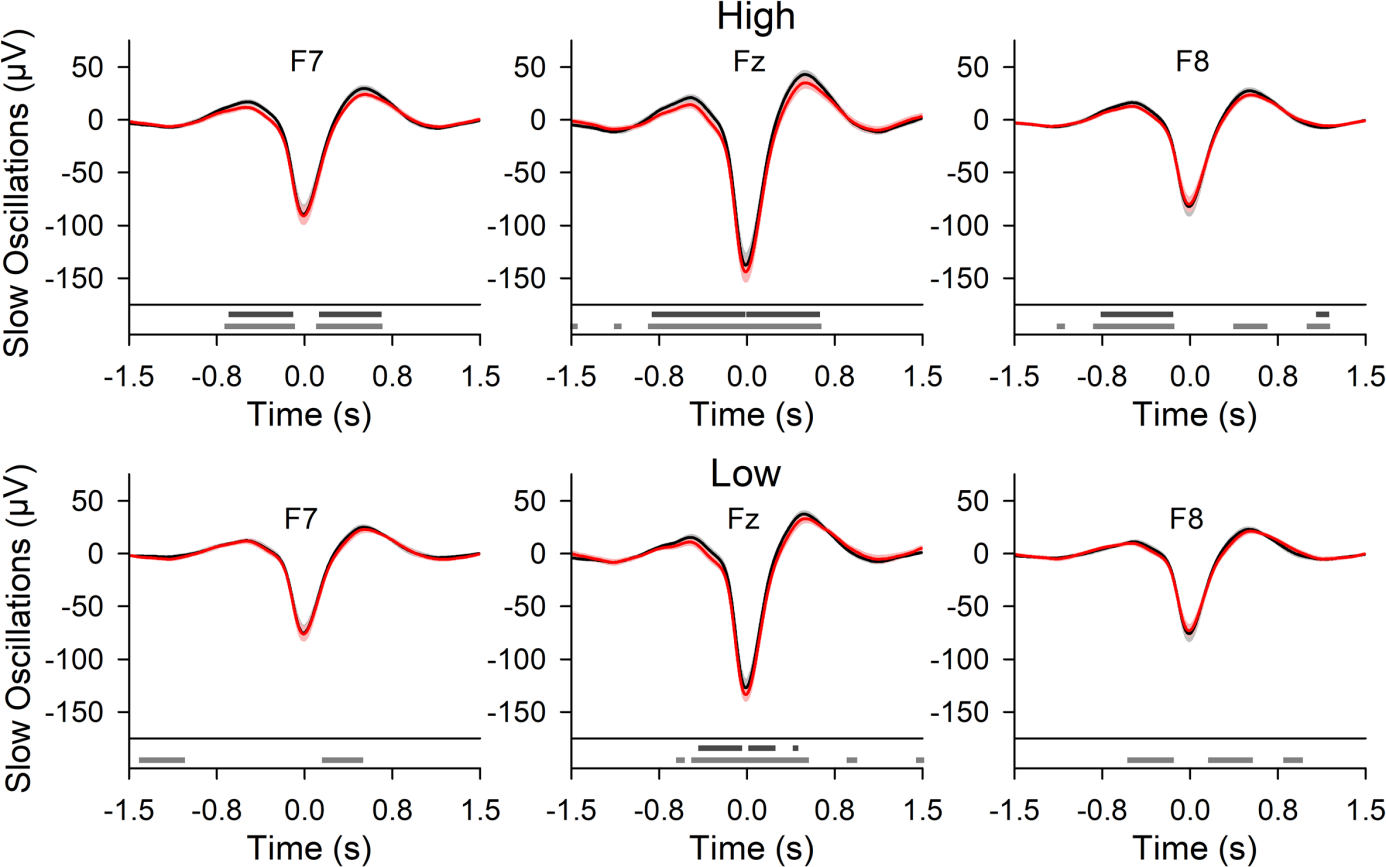
For all offline detected slow oscillations (SO) in the high and low APM group, the effect in CLAS and AmodCLAS. Top diagrams: Grand mean waveforms (± SEM) of spontaneous SO at locations F7, Fz, and F8 for CLAS (black) and AmodCLAS (red). Bottom diagrams: Horizontal bars indicate time points of significant differences. Wilcoxon signed‐rank test, without (gray) and with (dark gray) correction for false discovery rate, *p* < 0.05. High (*N* = 10) and low (*N* = 8) APM groups; median APM score is 110.

Interestingly, EEG modulations in offline detected SOs throughout NREM sleep, i.e., independent of acute acoustic stimulation delivery time, were slightly more pronounced in the high APM group (Figure 7). The effect of AmodCLAS on SOs detected during NREM sleep was topographically more widespread in the high APM group. Also, the up‐state positivity of the three frontal locations (F7, Fz, and F8) preceding and following the negative SO peak potential at ±0.3 to 0.6 s interacted significantly with the APM score (*F*(1,16) = 5.584, *p* = 0.031, for COND × APM; Table S2). For the high APM group, the up‐state positivity was significantly more negative in AmodCLAS than in CLAS (*Z* = −2.701, *p* = 0.007, *N* = 10). The low APM group also revealed a significant difference between conditions, but this difference failed to survive Bonferroni correction (*Z* = −2.100, *p* = 0.036, *N* = 8). In contrast to the spontaneous offline detected SO properties, stimulus‐related time‐locked responses and time‐locked spindle RMS were closely similar between the APM groups (Figures S6 and S7).

AmodCLAS also decreased sleep quality. This decrease was reflected across all subjects foremost by a decrease in the amount and percentage of time spent in REM sleep (*Z* > |−3.0|, *p* < 0.01, *N* = 20), increased time and percentage of WASO (*Z* > |−2.1|, *p* < 0.05), decreased sleep efficiency (*Z* = −2.203, *p* = 0.028), and a trend toward decreased TST (*Z* = −1.751, *p* = 0.08). An exploratory analysis of sleep architecture in the low and high APM groups separately indicated greater impairment of sleep in the low APM group. Only the decrease in the percentage of time spent in REM sleep was observed within both APM groups (*Z* > −2.1, *p* < 0.03; Table S1). To investigate more closely any relationship between APM score and sleep architecture, correlations with the architectural differences between groups were conducted. Only the difference between both conditions in the amount of time spent in REM sleep in the second night‐half correlated (negatively) with APM score (rho = −0.52, *p* = −0.018; Table S3 and Figure S8).

Investigations on the EEG frequency band power within NREM sleep revealed an overall suppression of power in AmodCLAS for the slow wave activity (main COND effect: *F*(1,19) = 4.516, *p* = 0.047), delta (*F*(1,19) = 6.093, *p* = 0.023), and theta bands (*F*(1,19) = 5.369, *p* = 0.032) with a trend in the same direction for the narrow SO band (*F*(1,19) = 4.077, *p* = 0.058; Figure S8). Interactions between condition and topography were not significant. However, following up on a statistical trend for the slow spindle frequency band revealed reduced power in AmodCLAS at F8 (*t*(19) = 2.250, *p* = 0.036; *F*(10,190) = 2.016, *p* = 0.093, for COND × TOPO interaction).

### 3.3 Psychometric Control Parameters

Subjects in both conditions rated higher on the positive affect scale in the morning than in the evening (*Z* > |−3.05|, *p* ≤ 0.002, for the difference between times). Similarly, subjects rated significantly lower (more awake) on the SSS in both groups (*Z* > |−3.58|; *p* < 0.001; Table S4). Morning scores on digit span and word fluency tests did not differ between conditions (*p* > 0.1).

We conducted an exploratory analysis on whether the individualized delay time from the EEG SO negative peak to the subsequent positive peak as well as the delta–theta ratio correlated with any behavioral measure, i.e., learning performance, psychometric parameter, or fluid intelligence. No correlation with either threshold was found, except, interestingly, a negative correlation between the delta–theta ratio and evening learning performance for both memory tasks (rho > − 0.5, *p* < 0.02).

## 4 Discussion

The aim of this study was to investigate whether tDCS during sleep could influence responses to CLAS, and to explore how such relatively modest changes in network excitability and activity might explain individual differences in responsiveness to CLAS. Correspondingly, AmodCLAS aimed to simulate, as compared with CLAS alone, a modest yet directionally defined change in brain state. We hypothesized that increasing frontocortical excitability and activity would alter behavior and sleep EEG in a unified manner, most likely to improve memory consolidation. Our hypothesis rested on the concept that an increase in cortical excitability would yield an increased sensitivity to hippocampal inputs related to reactivation (Wagner et al. 2010; Rothschild et al. 2017; Klinzing et al. 2019; Chao et al. 2020; Ngo, Fell, et al. 2020; Feliciano‐Ramos et al. 2023).

Across all subjects, AmodCLAS shifted offline detected SOs toward more negative values and increased SO length. Moreover, sleep efficiency was decreased, and notably, AmodCLAS reduced the time spent in REM sleep. Results did not reveal any overall effect on memory consolidation or postsleep learning by AmodCLAS. Interestingly, in a subsample of subjects scoring high on fluid intelligence, a measure of cognitive ability, memory consolidation on the NSWP task was significantly decreased as compared with CLAS. Concordantly, for subjects scoring high on fluid intelligence, electrophysiological differences were slightly more pronounced. We will discuss first the findings on memory consolidation and then on sleep architecture and electrophysiology.

Although cognitive ability was initially assessed as a control variable only, it appears that in our present population network activity, as defined by fluid intelligence, characterized responsiveness to AmodCLAS more strongly than the effects of anodal tDCS on responsiveness to CLAS. In those subjects, memory performance was impaired, contrary to our hypotheses.

An earlier study of ours with dorsolateral PFC anodal tDCS during slow‐wave sleep revealed a positive effect on memory consolidation (Marshall et al. 2004). A direct comparison is, however, limited: First, electrode sizes were significantly smaller, and the current density targeting the dorsolateral PFC (F3, F4), as well as the mastoid return electrodes, was significantly higher. Size and/or composition of the modulated network, with functional consequences for the excitation‐to‐inhibition balance and response threshold, therefore likely differed (Batsikadze et al. 2013; Adelhöfer et al. 2019; Antonenko et al. 2019; Koo‐Poeggel et al. 2019). Second, and more importantly, the effects of an external sensory stimulus on cortical processing related to memory consolidation were measured here. This is in contrast to the investigation into the modulatory effects of endogenous cortical activity (e.g., Marshall et al. 2004) as well as to investigations of corticofugal responses, as in tDCS–transcranial magnetic stimulation studies (e.g., Dissanayaka et al. 2017). CLAS is assumed to exert its beneficial effects on oscillatory coupling and consolidation processes by synchronizing neural events (Esfahani et al. 2023; Reith et al. 2025). In the present study, the specificity of task‐related neural reactivations may have been reduced if the hypothesized increase in neuronal excitability broadly engaged many nonspecific cortical networks. The relatively longer time window for the SO down‐state in AmodCLAS (cp. Figure S3) might be associated with a decreased likelihood of functionally relevant events induced by CLAS to synchronize with the SO up‐state. These considerations reveal an inherent caveat of our working hypotheses, namely, that a specific effect on hippocampal afferent activity, and not on corticofugal, cortical white matter, or even on deeper structures, cannot be ruled out (Reato et al. 2013; Chan et al. 2021; Katoch et al. 2023; Yachou et al. 2025). Finally, a detrimental effect on sleep by the combined stimulation procedures may have directly disturbed hippocampal reactivation (Giri et al. 2024). Regarding the subgroup scoring high on the APM test, increased susceptibility to the mild neuromodulation of AmodCLAS over the dorsolateral PFC could be dependent upon a different extent of neural efficiency (e.g., Ramchandran et al. 2019). During wakefulness, these authors reported that fluid intelligence predicted increased activation of the dorsal attention network to which the dorsolateral PFC belongs, although relationships between cognitive ability and fMRI‐derived brain activity under baseline conditions are under debate (Ferguson et al. 2017; Kruschwitz et al. 2018).

The reduction in REM sleep observed with AmodCLAS was entirely unexpected, despite a prior study indicating a trend toward decreased REM sleep duration with CLAS alone (Lustenberger et al. 2022) and reported reductions in NREM sleep N2 and N3 (Papalambros, Santostasi, et al. 2017; Papalambros, Weintraub, et al. 2019). Initial findings from neuroimaging studies on cerebral blood flow have mostly suggested an inactivation of dorsolateral PFC during REM sleep (Braun et al. 1997; Maquet et al. 2005). On this background, a simple explanation for the reduced time spent in REM sleep could be frontocortical activation by the anodal tDCS application. However, more recent studies point toward PFC activation during and its regulatory role in REM sleep (Kubota et al. 2011; Vijayan et al. 2017; Hong et al. 2023).

Interestingly, the overall REM sleep effect was linked to the APM score: Fluid intelligence correlated negatively with the REM sleep difference between conditions, with the higher scoring subjects showing a smaller decrease in REM sleep in AmodCLAS versus CLAS. Studies on REM sleep and cognitive ability are rare, yet a triad between REM sleep, fluid intelligence, and working memory appears to exist. REM sleep duration was found to increase after tasks requiring increased working memory load (Lau et al. 2015). Although functional associations between working memory and fluid intelligence were reported, their neural circuitries highly overlap (Kane and Engle 2002; Gray et al. 2003; Geiger et al. 2011). Therefore, we carefully interpret the relationship between APM and REM sleep duration in our study as an indication of greater neurophysiological resilience of the PFC and its REM sleep regulatory function. This greater resilience of the high APM group may appear to contradict the above‐mentioned increase in the presumed susceptibility of this group. Yet, we interpret the former as a trait‐like property more dependent upon “hard‐wiring” and the latter as a form of state‐dependent (short‐term) neuroplasticity.

The decrease in sleep efficiency with AmodCLAS falls in line with effects during waking, in which anodal tDCS increases cortical excitability and activation. The lack of poor sleep efficiency in previous studies may again be due to the aforementioned differences in stimulation parameters. One closely comparable study (Marshall et al. 2004) was terminated after the first NREM–REM sleep cycle, excluding assessment of sleep efficiency as well as REM sleep during the second half of the night.

Offline detected SOs throughout the night revealed the most consistent differences in EEG between conditions, across all subjects. We interpret the stronger modulation of spontaneous SO activity versus time‐locked responses as an indication that the specific sensory pathway was not primarily affected by AmodCLAS, but rather the spontaneous “background” activity. In the AmodCLAS condition, the up‐states of SOs bordering on the detected SO down‐state revealed, at fronto‐central topographies, prominent negative shifts. Spontaneous SOs were also of longer duration with a relatively stronger increase of negative relative to positive SO half‐wave length at the frontal midline location.

The SO period depends on the interplay between mechanisms of up‐state initiation, network refractory mechanisms during the down‐state, and probably intrinsic neuronal activity of deep‐layer pyramidal neurons. Particularly in the PFC up‐state, persistence is primarily due to synaptic activity involving NMDARs, recurrent excitatory synaptic activity, balanced by inhibition. Inhibitory input maintains the membrane potential close to firing threshold. The time during the down‐state at which sufficient activity has built up to initialize an up‐state depends on down‐state refractory mechanisms. Neuromodulatory tone strongly influences the level of activation and inactivation of activity‐dependent K+ conductances and short‐term synaptic depression, responsible candidates for the down‐state (Sanchez‐Vives and McCormick 2000; Timofeev et al. 2000; Bazhenov et al. 2002; Neske 2015).

Our reflections on the affected mechanisms leading to the offline detected SO changes in potential level and SO duration are: First, the negative shift in direction down‐state suggests AmodCLAS decreased the amount of persistent neural activity during the SO up‐phase. Second, the relative increase in the negative SO half‐wave length at frontal locations could likewise be explained by an offset of the excitation/inhibition balance in favor of an extended refractory period, i.e., down‐state. The shallower negative to positive SO slope with AmodCLAS also falls in line with a lower amount of excitation buildup during the (end of the) down‐state.

Of special interest is also the potential influence of AmodCLAS on neuromodulatory activity. Noradrenergic and cholinergic neuromodulatory systems are essentially involved in sleep regulation, and their activity can be influenced by tDCS (Muzur et al. 2002; Léna et al. 2005; Medeiros et al. 2012; Oh et al. 2022; Sulaman et al. 2024). Endogenous cholinergic activity, for instance, is low during SWS. A pharmacological increase in acetylcholine levels can impair sleep‐associated memory consolidation, which is attributed to a reduction in information flow from the hippocampus to the neocortex (Hasselmo 1999; Power 2004; Czarnecki et al. 2021). Endogenous noradrenergic levels are also low in SWS compared to waking, and minimal in REM sleep (Riemann et al. 1994). A phase‐dependent fine temporal interplay of noradrenergic locus coeruleus activity and sleep oscillations has been disclosed (Eschenko and Sara 2008; Novitskaya et al. 2016; Swift et al. 2018; Kjaerby et al. 2022). The locus coeruleus exerts activity on the PFC and hippocampus (e.g., Hansen 2017). Connections between brainstem neuromodulatory systems and the PFC are partly reciprocal (Arnsten and Goldman‐Rakic 1984; Zaborszky et al. 1997; Jodo et al. 1998; Sara 2009). Thus, it opens a window for AmodCLAS to (de)regulate systems of neural reactivation and consolidation beyond the PFC. Present findings and literature are too sketchy to predict directional effects; yet, it is important to emphasize that these chemical neuromodulators interact both with fluid intelligence (Tsukahara and Engle 2021) and SO properties, including up‐state duration (Lőrincz et al. 2015; Durán et al. 2021). In summary, independent of the exact mechanisms, the above underscores that AmodCLAS is in the position to change neuromodulatory tone and the balance of cortical excitation to inhibition, potentially influencing both sleep oscillatory EEG and sleep macrostructure. Systematic investigations into the influence of “hard‐wired” (brainstem neuromodulatory) neurochemistry on downstream neuroplastic events during sleep, and the ability of long‐term noninvasive brain stimulation to affect this relationship, present an attractive avenue for future sleep research.

In summary, AmodCLAS used here to simulate within‐subject shifts in brain state shifted the EEG toward negative potential values at frontal and increased SO duration at fronto‐central locations, reduced SO density, and modulated slow spindle duration. Overall, there was no effect on memory retention; however, in a subset of subjects with high fluid intelligence scores, a decrement in memory consolidation with AmodCLAS compared with CLAS was found.

A limitation of this study was the absence of a sham condition, spared for reasons of practicability, that would have allowed comparisons to basal network activity. Furthermore, because the goal was to deliver acoustic and electrical stimulation during N3 sleep, the timing was dependent on each subject's N3 duration, making occasional deviations unavoidable. Obviously, the rather small sample sizes of the APM groups warrant verification of results in future studies. Lastly, since the method of SO phase targeting has shown greater precision than the presently implemented CLAS method, it has yet to be investigated whether such a more precise algorithms for online SO detection (Ruch et al. 2022; Wunderlin et al. 2022) can overcome the variability observed for behavioral responses in SO closed‐loop studies.

## Author Contributions

**T. Hausdorf:** data curation, formal analysis, methodology, validation, writing – original draft, writing – review and editing. **A. Ferdinand:** data curation, formal analysis, methodology, validation, writing – original draft, writing – review and editing. **P. Koo‐Poeggel:** conceptualization, methodology, project administration. **M. Mölle:** conceptualization, formal analysis, methodology, writing – review and editing. **M. Bazhenov:** conceptualization, funding acquisition, writing – review and editing. **L. Marshall:** conceptualization, formal analysis, funding acquisition, methodology, project administration, supervision, validation, writing – original draft, writing – review and editing.

## Conflicts of Interest

The authors declare no conflicts of interest.

## Peer Review

The peer review history for this article is available at https://www.webofscience.com/api/gateway/wos/peer‐review/10.1111/ejn.70266.

## Supporting information


**Figure S1:** Violin plot for the distribution of APM scores for high and low APM groups.
**Figure S2:** Schematic of offline detected spontaneously occurring SOs during CLAS and AmodCLAS. Negative half‐waves indicate detected SOs in NREM sleep. A subgroup of SOs (red) occurred only during the acute tDCS‐on period (in AmodCLAS) or during the pseudo‐tDCS‐on phase (in CLAS). Corresponding properties are referred to as “All NREM” and “tDCS NREM” in Figure 4. *Note:* stimulus‐related responses are not indicated.
**Figure S3:** Duration of half‐waves (SOHW). Length of positive and negative slow oscillations of spontaneously detected SOs throughout NREM sleep. A thick circle indicates a main effect of condition in the corresponding repeated measures ANOVA (see main text). ^t^ *p* < 0.1, *p < 0.05, **p < 0.01; Wilcoxon test, two‐sided, uncorrected for multiple comparisons, *N* = 20.
**Figure S4:** Response to closed‐loop acoustic stimulation (CLAS) as compared with a true sham condition. The latter condition was not investigated in the present study. Grand mean waveforms (±SEM) of stimulus‐locked responses at Fz, Cz, and Pz referred to linked mastoids of CLAS (red line) and Sham stimulation (black line). Dashed lines indicate times of first and second acoustic stimulus delivery. Baseline normalization was from −0.1 to −0.8 s. Bottom diagrams: Black lines represent significance between CLAS and Sham corrected for a false discovery rate of 0.05; two‐sided *T*‐test for unequal variance. *N* = 15/16; data from one sham session were unobtainable (modified from Koo‐Poeggel et al. 2022).
**Figure S5:** Stimulus‐locked EEG responses to acoustic stimuli in CLAS and AmodCLAS. Top diagrams: Grand mean waveforms (± SEM) at locations F7, Fz, F8, C3, Cz, C4, P3, Pz, and P4 for CLAS (black) and AmodCLAS (red); baseline normalized data. Bottom diagrams: horizontal bars indicate time points of significant differences (Wilcoxon signed‐rank test, *p* < 0.05, uncorrected). For comparative purposes, since some earlier key studies on CLAS depicted uncorrected *p*‐values [1]. *N* = 19; for technical reasons, the triggers of one subject were not accessible. Baseline normalization occurred over −0.99 to −0.01 s. Averages per subject consisted of 275.1 ± 21.3 (228.4 ± 25.9) epochs for CLAS (AmodCLAS).
**Figure S6:** Stimulus‐locked responses to acoustic stimuli in CLAS and AmodCLAS for the High and Low APM groups. Top diagrams: Grand mean waveforms (± SEM) at locations F7, Fz, and F8 for CLAS (black) and AmodCLAS (red). Bottom diagrams: horizontal bars indicate time points of significant differences (Wilcoxon signed‐rank test, *p* < 0.05, uncorrected). Baseline normalization occurred over −0.99 to −0.01 s. *N* = 10 (*N* = 8) for the High (Low) APM group.
**Figure S7:** Stimulus‐locked fast spindle root mean square (RMS) to acoustic stimuli in CLAS and AmodCLAS for the High and Low APM groups. Top diagrams: Grand mean waveforms (± SEM) of stimulus‐locked responses at locations P3, Pz, and P4 for CLAS (black) and AmodCLAS (red). Bottom diagrams: Horizontal bars indicate time points of significant differences (Wilcoxon signed‐rank test, *p* < 0.05, uncorrected), *N* = 10.
**Figure S8:** Left: Percentage of time spent in REM sleep. *N* = 20 for Hours 2–7. For Hour 8: *N* = 19. For Hour 9: *N* = 10 (CLAS), *N* = 12 (AmodCLAS). Note for Hour 9, REM sleep occurred in only seven common subjects. Right: Nonparametric linear correlation between APM score and the difference in amount of time spent in REM sleep for AmodCLAS minus CLAS. *N* = 20.
**Table S1:** Sleep parameters for all subjects.
**Table S2:** Covariate analyses for offline detected SO‐positive intervals.
**Table S3:** Linear correlation between APM score and sleep differences between conditions.
**Table S4:** Psychometric control tests.
**Figure S7:** Heat maps of EEG power, averaged across CLAS and AmodCLAS (A), and as a difference in slow spindle EEG power between AmodCLAS and CLAS for the slow spindle band (B). SO, Slow oscillation, SWA, slow wave activity, SlSpi, slow spindle frequency band, FSpi, fast spindle frequency band.

## Data Availability

Data are accessible upon reasonable request.
