# Supplementary material for: Stimulating the Stimulated Cortex—Frontocortical Anodal Electric Stimulation Combined With Closed‐Loop Acoustic Stimulation During Sleep Impairs Memory in Subjects With High Cognitive Ability
Source: Eur J Neurosci. 2025 Oct 3;62(7):e70266. doi: 10.1111/ejn.70266 (PMC12493016; doi:10.1111/ejn.70266)
Supplement: Supplementary file 1 — Figure S1: Violin plot for the distribution of APM scores for high and low APM groups. Figure S2: Schematic of offline detected spontaneously occurring SOs during CLAS and AmodCLAS. Negative half‐waves indicate detected SOs in NREM sleep. A subgroup of SOs (red) occurred only during the acute tDCS‐on period (in AmodCLAS) or during the pseudo‐tDCS‐on phase (in CLAS). Corresponding properties are referred to as “All NREM” and “tDCS NREM” in Figure 4. Note: stimulus‐related responses are not indicated. Figure S3: Duration of half‐waves (SOHW). Length of positive and negative slow oscillations of spontaneously detected SOs throughout NREM sleep. A thick circle indicates a main effect of condition in the corresponding repeated measures ANOVA (see main text). t p < 0.1, *p < 0.05, **p < 0.01; Wilcoxon test, two‐sided, uncorrected for multiple comparisons, N = 20. Figure S4: Response to closed‐loop acoustic stimulation (CLAS) as compared with a true sham condition. The latter condition was not investigated in the present study. Grand mean waveforms (±SEM) of stimulus‐locked responses at Fz, Cz, and Pz referred to linked mastoids of CLAS (red line) and Sham stimulation (black line). Dashed lines indicate times of first and second acoustic stimulus delivery. Baseline normalization was from −0.1 to −0.8 s. Bottom diagrams: Black lines represent significance between CLAS and Sham corrected for a false discovery rate of 0.05; two‐sided T‐test for unequal variance. N = 15/16; data from one sham session were unobtainable (modified from Koo‐Poeggel et al. 2022). Figure S5: Stimulus‐locked EEG responses to acoustic stimuli in CLAS and AmodCLAS. Top diagrams: Grand mean waveforms (± SEM) at locations F7, Fz, F8, C3, Cz, C4, P3, Pz, and P4 for CLAS (black) and AmodCLAS (red); baseline normalized data. Bottom diagrams: horizontal bars indicate time points of significant differences (Wilcoxon signed‐rank test, p < 0.05, uncorrected). For comparative purposes, since some earlier key stu [file EJN-62-0-s001.pdf]

Supplementary information

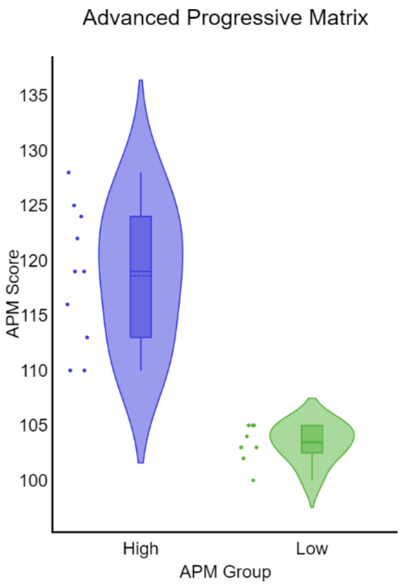

Supplementary Figure 1
Violin plot for the distribution of APM scores for high and low APM groups.

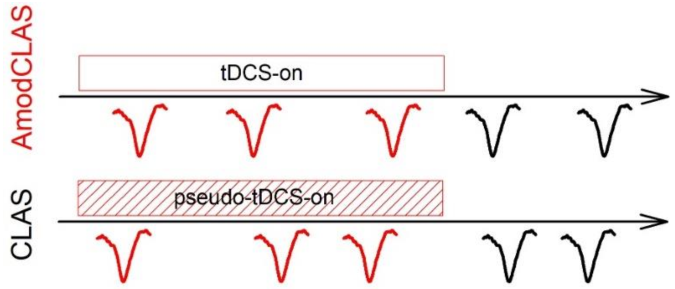

Supplementary Figure 2
Schematic of offline detected spontaneously occurring SOs during CLAS and AmodCLAS. Negative half-waves indicate in NREM sleep detected SOs. A subgroup of SOs (red) occurred only during the acute tDCS-on period (in AmodCLAS) or during the pseudo-tDCS-on phase (in CLAS). Corresponding properties are referred to as 'All NREM' and 'tDCS NREM' in Figure 4. Note, stimulus-related responses are not indicated.

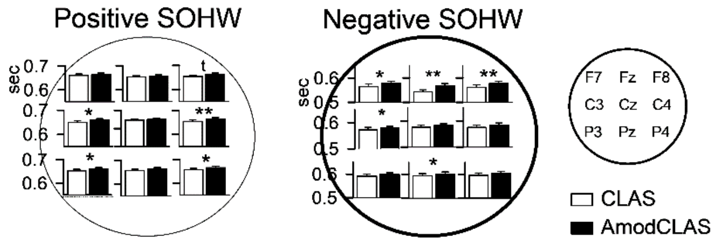

Supplementary Figure 3
Duration of half-waves (SOHW). Length of positive and negative slow oscillations of spontaneously detected SOs throughout NREM sleep. Thick circle indicates a main effect of condition in the corresponding repeated measures ANOVA (see main text). ^{t} p < 0.1, * p<0.05, ** p< 0.01; Wilcoxon test, two-sided, uncorrected for multiple comparisons, N = 20.

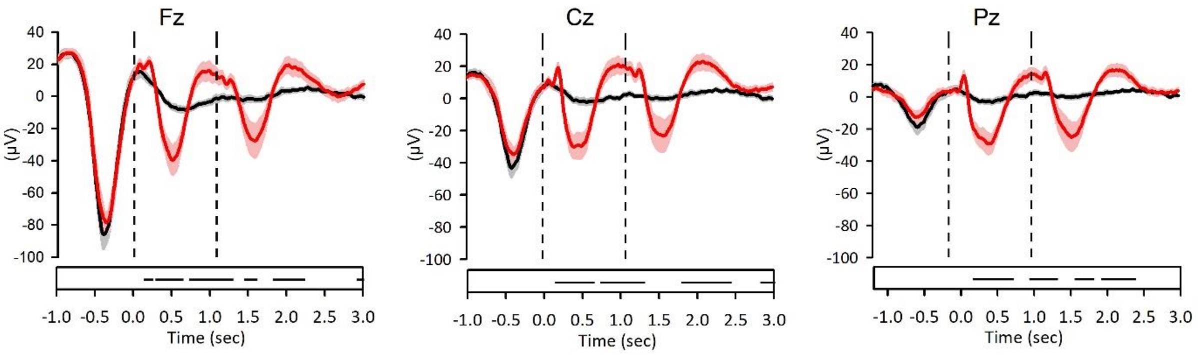

Supplementary Figure 4
Response to closed-loop acoustic stimulation (CLAS) as compared to a true sham condition. The latter condition was not investigated in the present study. Grand mean waveforms (± SEM) of stimulus-locked responses at Fz, Cz and Pz referred to linked mastoids of CLAS (red line) and Sham stimulation (black line). Dashed lines indicate times of first and second acoustic stimulus delivery. Baseline normalization was from -0.1 to -0.8 s. Bottom diagrams: Black lines represent significance between CLAS and Sham corrected for a false discovery rate of 0.05; two-sided T-test for unequal variance. N = 15/16; data of one sham session were unobtainable (modified from Koo-Poeggel et al., 2022).

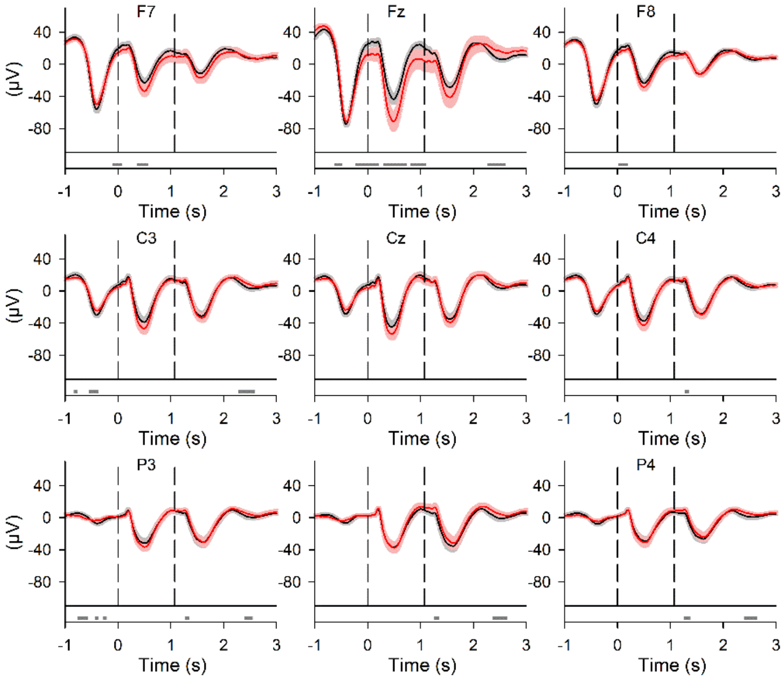

Supplementary Figure 5
Stimulus-locked EEG responses to acoustic stimuli in CLAS and AmodCLAS. Top diagrams: Grand mean waveforms (± SEM) at locations F7, Fz, F8, C3, Cz, C4, P3, Pz and P4 for CLAS (black) and AmodCLAS (red); baseline normalized data. Bottom diagrams: horizontal bars indicate time points of significant differences (Wilcoxon signed-rank test, $p < 0.05$, uncorrected). For comparative purposes, since some earlier key studies on CLAS depicted uncorrected p-values [1]. N = 19, for technical reasons the triggers of one subject were not accessible. Baseline normalization occurred over -0.99 to -0.01 sec. Averages per subject consisted of 275.1±21.3 (228.4±25.9) epochs for CLAS (AmodCLAS).

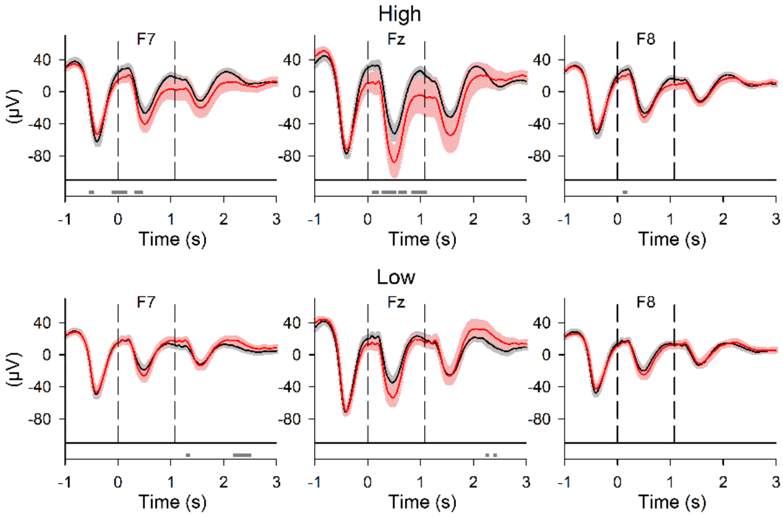

Supplementary Figure 6
Stimulus-locked responses to acoustic stimuli in CLAS and AmodCLAS for the High and Low APM group. Top diagrams: Grand mean waveforms (± SEM) at locations F7, Fz and F8 for CLAS (black) and AmodCLAS (red). Bottom diagrams: horizontal bars indicate time points of significant differences (Wilcoxon signed-rank test, $p < 0.05$, uncorrected). Baseline normalization occurred over -0.99 to -0.01 sec. N = 10 (N = 8) for the High (Low) APM group.

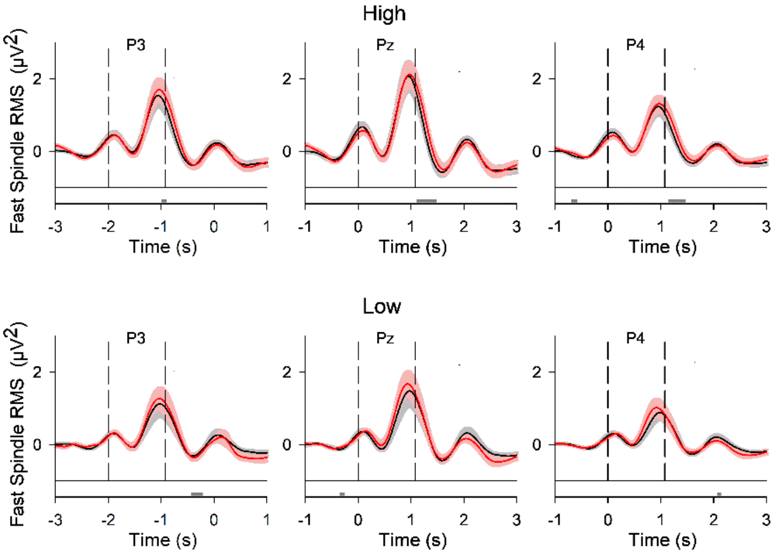

Supplementary Figure 7
Stimulus-locked fast spindle root mean square (RMS) to acoustic stimuli in CLAS and AmodCLAS for the High and Low APM groups. Top diagrams: Grand mean waveforms (± SEM) of stimulus-locked responses at locations P3, Pz and P4 for CLAS (black) and AmodCLAS (red). Bottom diagrams: horizontal bars indicate time points of significant differences (Wilcoxon signed-rank test, $p < 0.05$, uncorrected), N = 10.

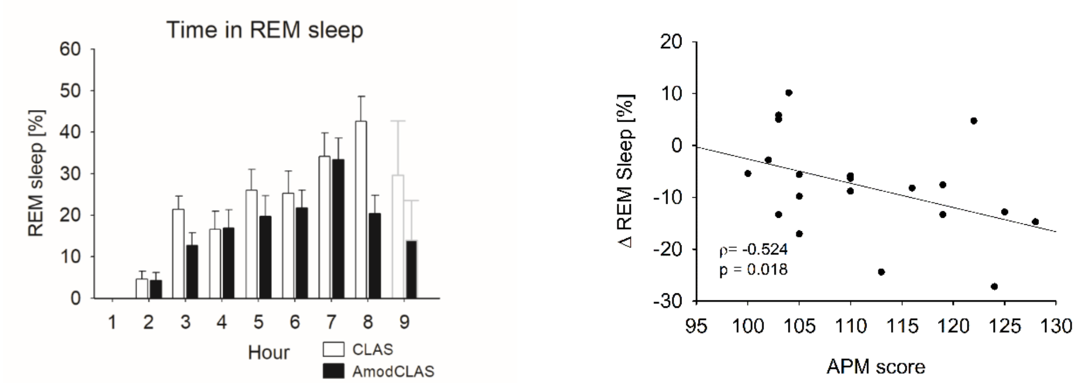

Supplementary Figure 8
Left: Percentage of time spent in REM sleep. N = 20 for hours 2-7. For hour 8: N = 19. For hour 9: N = 10 (CLAS), N = 12 (AmodCLAS). Note for hour 9 REM sleep occurred in only 7 common subjects. Right: Non-parametric linear correlation between APM score and the difference in amount of time spent in REM sleep for AmodCLAS minus CLAS. N = 20.

Supplementary Table S1

Sleep parameters for all subjects

	**CLAS**	**AmodCLAS**
TST (min)	441.6±5.7	429.6±7.2 ^{t}
WASO (%)	6.1±0.8	9.6±1.3 *
N1 (%)	4.2±0.4	5.7±1.0
N2 (%)	47.5±1.4	49.2±1.7
N3 (%)	21.6±1.4	19.6±1.6^{t}
REM sleep (%)	20.7±1.0	15.9±1.0 **
SE	93.9±0.8	90.0±1.3 *

* p<0.05, ** p< 0.01; Wilcoxon test, two-sided, uncorrected for multiple comparisons, N = 20.

Sleep parameters of the Low APM Group (N = 8)

	**CLAS**	**AmodCLAS**	**Z**	**p-value**
TST (min)	449.4±6.6	436.5±6.8	-2.028	.043
WASO (%)	5.6±0.9	8.5±1.4	-2.100	.036
N1 (%)	4.1±0.7	6.1±1.2		
N2 (%)	49.2±1.2	50.1±2.9		
N3 (%)	20.5±2.0	19.1±2.7	-.700	.484
REM sleep (%)	20.6±1.5	16.2±1.4	-2.380	.017
SE	94.4±0.9	91.5±1.4	-2.100	.036

Only data of subjects corresponding to the Low APM Group in the NSWP task are shown.

Sleep parameters of the High APM Group (N = 10)

	**CLAS**	**AmodCLAS**	**Z**	**p-value**
TST (min)	437.2±10.0	419.7±12.4	-1.172	.241
WASO (%)	6.4±1.3	11.5±2.1	-1.580	.114
N1 (%)	4.3±0.7	6.2±1.6		
N2 (%)	45.7±2.5	47.1±2.2		
N3 (%)	22.9±2.0	20.6±2.3	-1.376	.169
REM sleep (%)	20.7±1.8	14.6±1.4	-2.191	.028
SE	93.6±1.3	88.5±2.1	-1.580	.114

Only data of subjects corresponding to the High APM Group in the NSWP task are shown.

Supplementary Table S2 Covariate analyses for offline detected SO positive intervals

	F-value	p-value
Cond (1,16)	3.855	.067
Cond*APMscore (1,16)	5.584	.031

Supplementary Table S3 Linear correlation between APM score and sleep differences between conditions

	Coefficient	p-value
ΔREM sleep (%)	-0.133	0.576
ΔWASO (%)	0.163	0.492
ΔREM sleep (5-8h) **%**	-0.524	0.018*
ΔSE (%)	-0.163	0.492

Δ, Difference for AmodCLAS minus CLAS**,** * $p < 0.05$; Spearmans rho, two-sided, uncorrected for multiple comparisons, N = 20.

Supplementary Table S4 Psychometric control tests

	**CLAS**		**AmodCLAS**	
	Evening	Morning	Evening	Morning
SSS	4.2±0.3	2.4±0.2 **	4.4±0.3	2.3±0.2 **
PANAS-P	2.6±0.2	3.1±0.1 **	2.5±0.2	3.1±0.2 **
PANAS-N	1.1±0.0	1.1±0.0	1.1±0.0	1.1±0.0

** $p < 0.01$; Wilcoxon test, two-sided, uncorrected for multiple comparisons; SSS, Stanford Sleepiness Scale; PANAS, Positive and Negative Affect scale; N = 20

A

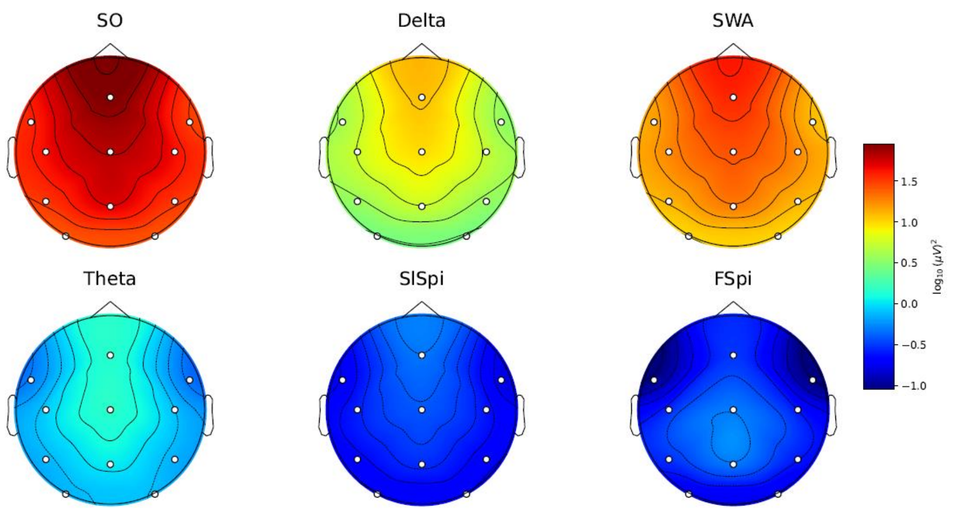

B

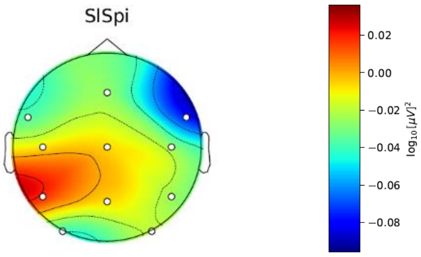

Supplementary Figure 7

Heat maps of EEG power, averaged across CLAS and AmodCLAS (A) and as a difference in slow spindle EEG power between AmodCLAS and CLAS for the slow spindle band (B). SO, Slow oscillation, SWA, slow wave activity, SlSpi, slow spindle frequency band, FSpi, fast spindle frequency band.

[1] Ngo, HV, Claussen, JC, Born, J & Mölle, M. (2013) Induction of slow oscillations by rhythmic acoustic stimulation. J Sleep Res, 22, 22-31.

Ngo, HV, Miedema, A, Faude, I, Martinetz, T, Mölle, M & Born, J (2015) Driving sleep slow oscillations by auditory closed-loop stimulation-a self-limiting process. J Neurosci, 35, 6630-6638.

Ong, JL, Lo, JC, Chee, NI, Santostasi, G, Paller, KA, Zee, PC & Chee, MW (2016) Effects of phase-locked acoustic stimulation during a nap on EEG spectra and declarative memory consolidation. Sleep Med, 20, 88-97.
